# Effects of Nano-CaCO_3_ Content on the Crystallization, Mechanical Properties, and Cell Structure of PP Nanocomposites in Microcellular Injection Molding

**DOI:** 10.3390/polym10101160

**Published:** 2018-10-17

**Authors:** Huajie Mao, Bo He, Wei Guo, Lin Hua, Qing Yang

**Affiliations:** 1 School of Materials Science and Engineering, Wuhan University of Technology, Wuhan 430070, China; maohj_whut@163.com (H.M.); hbwhut@163.com (B.H.); 2 Hubei Key Laboratory of Advanced Technology for Automotive Components, Wuhan University of Technology, Wuhan 430070, China; hualin@whut.edu.cn (L.H.); yq941210@163.com (Q.Y.); 3 Hubei Collaborative Innovation Center for Automotive Components Technology, Wuhan 430070, China; 4 School of Automotive Engineering, Wuhan University of Technology, Wuhan 430070, China

**Keywords:** microcellular injection molding, nano-calcium carbonate, crystallization, cell structure, mechanical properties

## Abstract

Using supercritical nitrogen as the physical foaming agent, microcellular polypropylene (PP) nanocomposites were prepared in microcellular injection molding. The main purpose of this work is to study effects of content of nano-CaCO_3_ on the crystallization, mechanical properties, and cell structure of PP nanocomposites in microcellular injection molding. The results show that adding nano-CaCO_3_ to PP could improve its mechanical properties and cell structure. The thermal stability and crystallinity enhances with increase of nano-CaCO_3_. As a bubble nucleating agent, adding nano-CaCO_3_ to PP improves the cell structure in both the parallel sections and vertical sections. The mechanical properties increase first and then decrease with increase of nano-CaCO_3_. The mechanical properties are affected by the cell structure, as well. The mechanical properties and cell structure are optimum when the content of nano-CaCO_3_ is 6 wt %.

## 1. Introduction

Polypropylene (PP) has been widely used in many fields, such as in electrical equipment, furniture, and automobile interiors. In the automobile interiors, reducing the amounts of polymer is an important way to lightweight. Also, microcellular foam is an advanced technology used to reduce the amount of polymer used. Microcellular foam can make the polymer create a cell structure, which has a cell diameter less than 10 μm and a cell density larger than 10^9^ cells/cm^3^. Microcellular foam polymers are new materials with a high impact strength, high insulation properties, and low electrical conductivity. However, there are some disadvantages to using microcellular foam products, such as a decrease of the tensile strength, bad surface quality, and an uneven distribution of cells.

For improving the performance and cell morphology of microcellular foam products, adding nano-filler as nucleation agents to the polymer was widely used by the researchers. On the one hand, the nano-filler additives could improve the distribution of the cells. The fine cells distribution, cell size, and cell density greatly affected the mechanical properties. Marsavina et al. found that the fracture toughness increased with increase of the foam density, and the yield strength improved because of a higher foam density [1,2]. On the other hand, at the reinforcement phase, the addition of nano-filler improves the mechanical properties. There are various kinds of nano-filler that are added into microcellular polymer as nucleating agents, such as nano-clay, nano-silica, nano-CaCO_3_, multiwalled carbon nanotube, and so on. Nano-clays are widely used because of their good economic benefits. For example, Hwang added the montmorillonite into the polybutylene terephthalate (PBT), and the results show that the addition of montmorillonite improved the thermal stability of the microcellular injection molded PBT/clay nanocomposites, and the optimum content of organoclay was 1.0 wt % [3]. The research indicates that the influence of montmorillonite on the mechanical and rheological properties of microcellular injection ethylene-vinyl acetate copolymer (EVA)-nanocomposties and that the mechanical properties increased as the clay content increased [4]. Polylactide (PLA) is an environment friendly polymer that can be biodegradable in the natural environment, but its low melt strength restricts its application. Adding nano-clay into PLA is a common way to improve its melt strength. For example, Naqi et al. added organic modified nano-clay into liner and branched PLA, and they found that the addition of nano-clay is good for improving the crystallization behavior and morphology of foams [5]. Xie et al. found that the addition of 5 wt % nano-clay significantly improved the cell structure, mechanical properties, and surface quality [6]. Layered-silicate is also a common additives because of its unique layered structure. Researchers found that the exfoliated layered-silicate performed better in improving the nucleation efficiency of microcellular foam polystyrene (PS) than the aggregated layered-silicate [7,8]. Regarding the effect of particle size on cells, Zakiyan et al. found that the cell size decreases and the cell density increases with the decrease of the nano-silica size [9]. The low electrical conductivity of the microcellular polymer will restrict its implication. Ameli et al. found that adding multiwalled carbon nanotubes (MWCNT) could improve the electrical conductivity of microcellular PP, which can be used in electromagnetic shielding [10]. As for nano-CaCO_3_, a common additive, there are many studies about the influence of nano-CaCO_3_ on the microcellular polymer. X shi et al. found that, with addition of nano-CaCO_3_ into a microcellular PLA/PBAT blend, the cells present a significant increase in the cell uniformity and cell density [11]. Ding et al. investigated the foaming behavior of microcellular foam polypropylene/modified nano-calcium carbonate composites and found that the adjustment of the processing condition could improve the cell structure and mechanical properties [12]. Ding also investigated the contents of nano-CaCO_3_ on the microcellular PP, and a batch process was used. The results show that PP/5 wt % nano-CaCO_3_ exhibits an optimum cell structure [13]. Huang investigated the cell structure of the foamed nano-CaCO_3_/PP in a batch process, too [14]. Yu investigated the PS/nano-CaCO_3_ foams using an extrusion foaming process, and results showed that the nano-CaCO_3_ affected the cell sizes significantly [15]. In this work, we used microcellular injection molding to foam the composites, because this process is more convenient and efficient.

Therefore, the nano-fillers affect the crystallization behavior, mechanical properties, and cell structure badly. However, there are no detailed studies about the cell structure and the effect of the cell structure on mechanical properties. In this work, we reported experimental studies to systematically investigate the effects of the nano-CaCO_3_ content on the crystallization behavior, cell structure, and mechanical properties of the microcellular foamed PP. The cells of different sections and layers are discussed, respectively.

## 2. Experimental

### 2.1. Material

Polypropylene (K8303) with a melt flow index of 2 g/10 min was supplied by Sinopec Beijing Yanshan Co., Ltd. (Beijing, China). The nano-CaCO_3_ with a mean particle diameter of 60–80 nm was supplied by Changshan Jinxiong Co., Ltd. (Changshan, China). The PP-g-mah (1.2% grafting) that was used as a compatilizer was provided by Dongyuan Ziheng Plastics Co., Ltd. (Dongguan, China). The industrial N_2_ (99% purity) that was used as a foaming agent was provided by Wuhan XiangYun Industry Co., Ltd. (Wuhan, China).

### 2.2. Preparation of Nanocomposites

**Figure 1:**
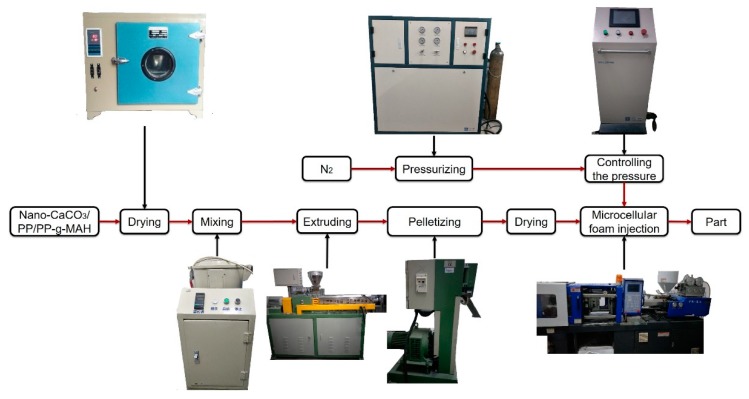
The process flow chart.

The microcellular injection molding process is shown in Figure 1. During the preparation of the nanocomposites, the materials were dried using a drying oven (101A-1, Guangdi, Instrument Equipment Co., Ltd., Shanghai, China), mixed using a mixer (SHR-10, Yiyang Plastic Machinery Co., Ltd., Wuhan, China), extruded using a twin-screw extruder (SHJ-20, Giant Machinery Co., Ltd., Nanjing, China), and pelletized using a pelletizer (LQ-20, Giant Machinery Co., Ltd., Nanjing, China).

The nano-CaCO_3_ contents of the composites were 2, 4, 6, 8, and 10 wt %, respectively. The 5 wt % PP-g-mah was added into all of the composites. The materials were dried for 24 h at 80 °C. The temperature of the barrel of the extruder was 190–190–190–200–200 °C.

### 2.3. Foaming Process

As shown in Figure 1, during the foaming process, the industrial nitrogen was a supercritical fluid (SCF) after being pressurized over 28 MPa through a pressure pump (GBL-200/350, CHN-Top Machinery Group Co., Ltd, Beijing, China). The barrel temperature of the injection molding machine, HDX50, (Haida Plastic Machinery Co., Ltd, Ningbo, China) was 200–210–210–200 °C. The pressure of SCF-N_2_ was controlled by a microcellular foaming console, (CHN-Top Machinery Group Co., Ltd, Beijing, China). The supercritical fluid N_2_ (17 MPa) was injected into the barrel when the composites were plasticized. The injection time of SCF-N_2_ was 3 s, and the content of SCF-N_2_ was 0.5%. The nano-composites and SCF-N_2_ changed to the single phase melt after the mixing function of the screw. The injection pressure of the injection machine was 80 MPa and the back pressure was 10 MPa. The nano-composites foamed after the melt was injected in the mold, because of the decrease in the pressure and temperature. The samples were completed after cooling for 20 s.

### 2.4. Characterizations

A DSC8500 (PE, Waltham, MA, USA) was used for the differential scanning calorimeter (DSC) analysis, and the analysis was done using two heating cycles over a temperature range of 40–200 °C in the heating/cooling rate of 10 °C/min. The tests were performed in an atmosphere of N_2_.

A thermogravimetric analysis (TGA) was performed with a STA2500 (Netzsch, Würzburg, Germany), in the temperature range of 40–800 °C, at the heating speed of 10 °C/min in an atmosphere of N_2_.

**Figure 2:**
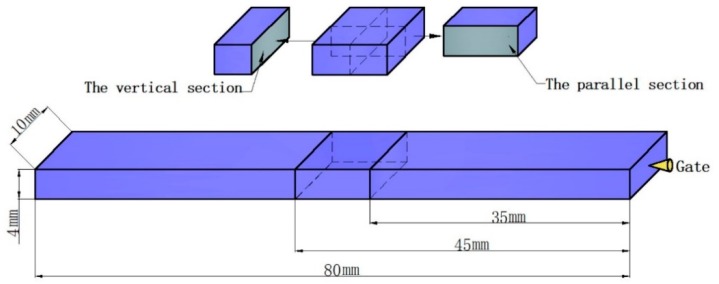
Preparation of the vertical and parallel sections from the flexural sample.

A JSM-IT300 (JEOL Ltd., Tokyo, Japan) scanning electron microscope (SEM) was used to characterize the cell structure and the distribution of the cells. The flexural samples were immersed in liquid nitrogen for 3 h. The flexural samples were fractured, and the fractured surface was coated with a gold layer before SEM testing. The parallel and vertical sections (10 mm × 4 mm) were taken from the flexural samples, as shown in Figure 2.

The average cell diameter could be calculated with the following equation:

$$\;D=\frac{\sum_{i=1}^{n}d_{i}}{n}\;$$

$d_{i}\;$is the cell diameter of a single cell in the given area, and *n* is the number of cells. The density of the cell can be calculated with the following equation:

$$\;N={\left(\frac{n\times M^{2}}{A}\right)}^{\frac{3}{2}}\;$$

*M* is the magnification of SEM, and *A* is the area of the picture.

**Figure 3:**
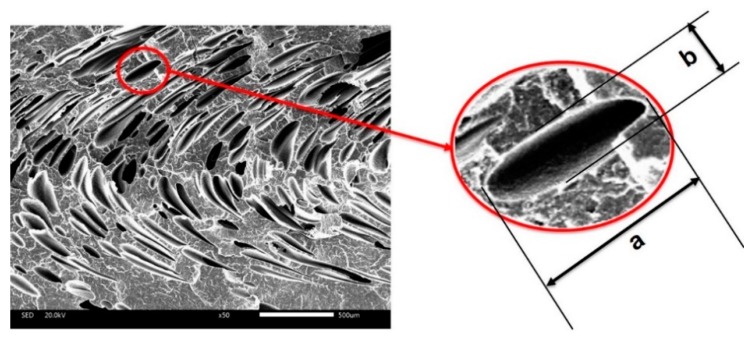
The length and diameter of a cell.

As for the parallel section, the transition layer cells that nucleated and grew at the filling time deformed, and turned into an irregular shape. The mean ratio of length–diameter of the cells was used to describe the degree of deformation. The length and diameter of a cell are shown in Figure 3, as follows:

As shown in Figure 3, the ratio of length–diameter can be calculated by the following equation: c = a/b. It can easily be concluded that the ratio of length–diameter will decrease with the decrease of deformation.

An electromechanical universal test machine, CMT6104, (MTS Systems Corp. Eden Prairie, MN, USA) was used to measure the tensile properties and flexural properties. The method for the tensile tests was ISO 527-1:1993, and the crosshead speed was 50 mm/min. The method for the flexural tests was ISO 178:2001, and the speed was 2 mm/min. The impact strength (IZOD) was obtained according to ISO 180:2000. The values of all of the mechanical properties were calculated using the average values of five specimens.

## 3. Results and Discussion

### 3.1. Effect of the Content of Nano-CaCO_3_ on the Crystallization Behaviour

#### 3.1.1. Crystallization and Melting

**Figure 4:**
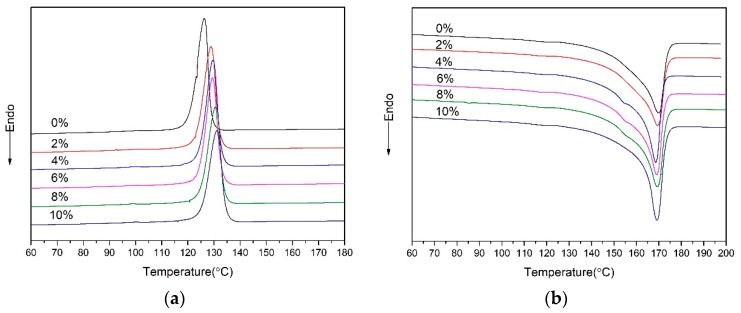
The differential scanning calorimeter (DSC) curves of nanocomposites: (**a**) crystallization curves; (**b**) melting curves.

The results of the DSC are shown in the Figure 4, and it can be found that the crystallization temperature increased with the addition of nano-CaCO_3_. The reason is that, as a nucleating agent, nano-CaCO_3_ reduced the degree of supercooling. With the addition of nano-CaCO_3_, the main method of nucleating the nanocomposites was heterogenous nucleation. As for the melt curves, the melt peak temperature had no obvious change with increase of nano-CaCO_3_. When the content of nano-CaCO_3_ was 4, 6, and 8%, a tiny peak existed around 154 °C, and it was a fusion peak of β-crystal. It indicated that the proper addition of nano-CaCO_3_ could promote β-crystal generating. β-crystal in matrix could improve the toughness of the PP. A dominant peak existed at 170 °C, it was a fusion peak of α-crystal. As shown in Table 1, when the crystallinity increased, it could indicate that the nano-CaCO_3_ addition also promoted crystallization. 

Crystallization (X_c_) can be calculated by the following equation:

$$X_{c}(\%)\;=\;\frac{\Delta H_{m}}{\Delta H}\;\times 100$$

where $\Delta H_{m}$ is the heat of fusion, and ΔH is the heat of fusion for 100% crystalline PP (209 J/g for α-PP). The melt peak temperature (T_m_), crystallization temperature (T_c_), heat of fusion (H_m_), and crystallization (X_c_) of the nanocomposites are compared in the Table 1. The rules for how T_m_ and T_c_ change have been discussed above. The H_m_ and crystallinity increased with increase of nano-CaCO_3_. As a nucleating agent, the addition of the nano-CaCO_3_ improved the efficiency of crystal, and provided more nucleating sites. For the nano-CaCO_3_ with more than 6 wt %, the increment of crystallinity decreases, as shown in Table 1. As a result of nano-CaCO_3_ conglomerating, the efficiency of the nucleating agent declines. The crystallinity affects the mechanical properties. So, the addition of nano-CaCO_3_ could improve the material’s hardness and elastic modulus [16].

#### 3.1.2. Thermogravimetric Analysis

**Figure 5:**
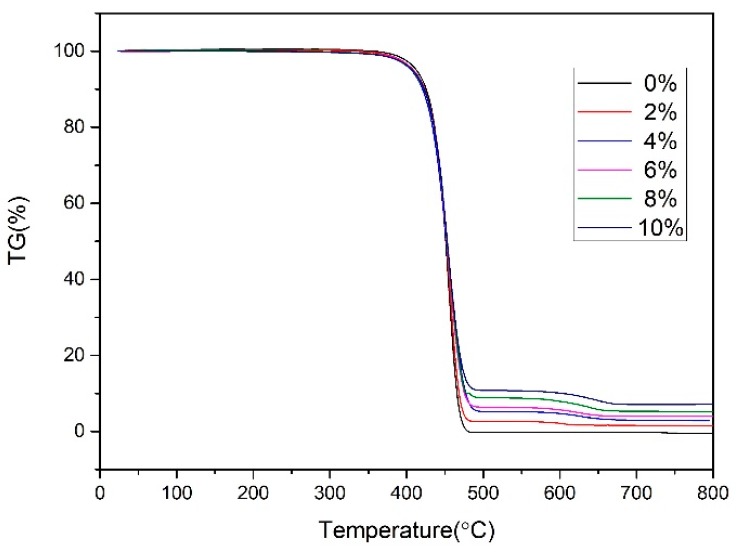
Thermogravimetric analysis (TGA) curves of nanocomposites.

The results of TGA are shown in Figure 5, and it can be seen that there is residue at 800 °C when adding the nano-CaCO_3_ into the composites. There were two decomposition stages of nanocomposites. In the first stage, the PP and compatilizer started decomposing at 400 °C. In the second stage, the nano-CaCO_3_ started decomposing at 600 °C.

Table 2 shows the detailed data of the TGA. The addition of nano-CaCO_3_ had little effect on the decomposition temperature (T_d_). However, if the differential thermal gravity (DTG) increased with the increase of nano-CaCO_3_, it implied that the thermal stability increased with the increase of nano-CaCO_3_. At 550 °C, the polymer matrix almost completed its decomposition, and the residue was nano-CaCO_3_. This indicated that the content of nano-CaCO_3_ of the composites is almost same as the formula. Nano-CaCO_3_ started to decompose into CO_2_ and CaO at around 600 °C.

### 3.2. Effect of the Content of Nano-CaCO_3_ on the Mechanical Properties

With the addition of nano-CaCO_3_, it is obvious that the mechanical properties will change. The microcellular injection molding processing also has an effect on the mechanical properties. It is meaningful to investigate how the addition of nano-CaCO_3_ and the microcellular foam processing affect the mechanical properties of the samples.

#### 3.2.1. Tensile Properties

**Figure 6:**
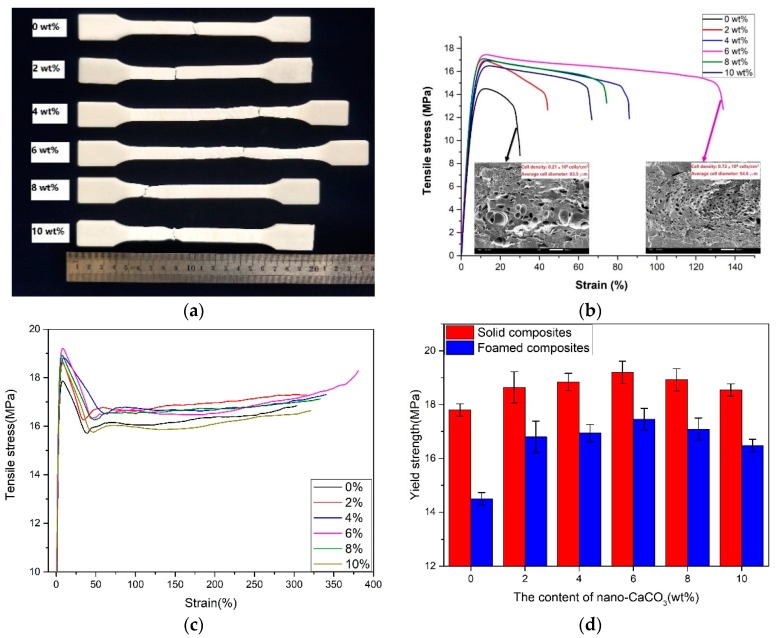
The results of the tensile tests are as follows: (**a**) the foamed samples after tensile testing; (**b**) the tensile stress–strain curves of foamed composites; (**c**) the tensile stress–strain curves of solid composites; and (**d**) the yield strength.

As shown in Figure 6, it can be concluded that the tensile properties (elongation at break and yield strength) of the samples increased with the increasing nano-CaCO_3_, and decreased as the content of nano-CaCO_3_ was over 6 wt %. When the content of nano-CaCO_3_ was 6 wt %, the yield strength was 17.5 MPa, and the elongation at break was 134%. The tensile stain–stress curves of the solid composites and foamed composites were totally different, as shown in the Figure 6b,c, and the solid samples were tougher. On the one hand, the yield strength of the samples with larger cells was smaller than that of the samples with smaller cells, as shown in the Figure 6b, because the effective loading area of the sample with the larger cells was smaller than the one with smaller cells in the period of the tensile test. The sample with the small cells can resist the propagation of cracks effectively, thus improving the tensile properties. On the other hand, as shown in the Figure 6d, the addition of nano-CaCO_3_ could improve the mechanical properties of solid polymer [17,18]. The tensile of the foamed composites decreased compared with the solid composites. But when the content of nano-CaCO_3_ was 6 wt %, the decrement was the smallest. With the addition of nano-CaCO_3_, the cell structure and distribution improved, because of the heterogeneous nucleation. The fine cell structure was advantageous for improving the yield strength. It can be concluded that both the cell structure and nano-CaCO_3_ affected the yield strength. As the content of nano-CaCO_3_ was over 6 wt %, the nano-CaCO_3_ conglomerated. The improvement of nano-CaCO_3_ on the tensile properties decreased.

#### 3.2.2. Flexural Properties

**Figure 7:**
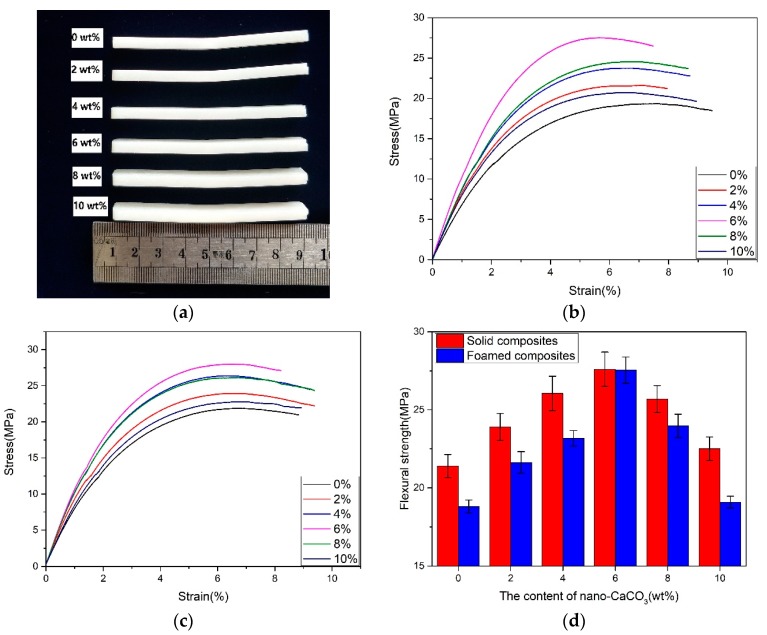
The results of the flexural tests are as follows: (**a**) the foamed samples after flexural testing; (**b**) the flexural strain–stress curves of the foamed composites; (**c**) the flexural strain–stress curves of the solid composites; (**d**) the flexural strength.

As shown in Figure 7b,c, the flexural strain–stress curves of the solid and foamed composites were similar. The addition of nano-CaCO_3_ could improve the flexural strength of both the solid and foamed composites. The flexural strength increased first, and then decreased, with the addition of nano-CaCO_3_. The improvement of the flexural strength solid composites means that the rigidity of nano-CaCO_3_ could improve the stiffness. However, the flexural strength of the foamed composites was smaller than that of the solid composites. The decrement of the flexural strength was the smallest, because the fine cell structure could improve the flexural strength. As the content of nano-CaCO_3_ was 8 and 10 wt %, the nano-CaCO_3_ conglomerated and cell structure got worse, so the flexural strength of the foamed composites decreased.

#### 3.2.3. Impact Properties

**Figure 8:**
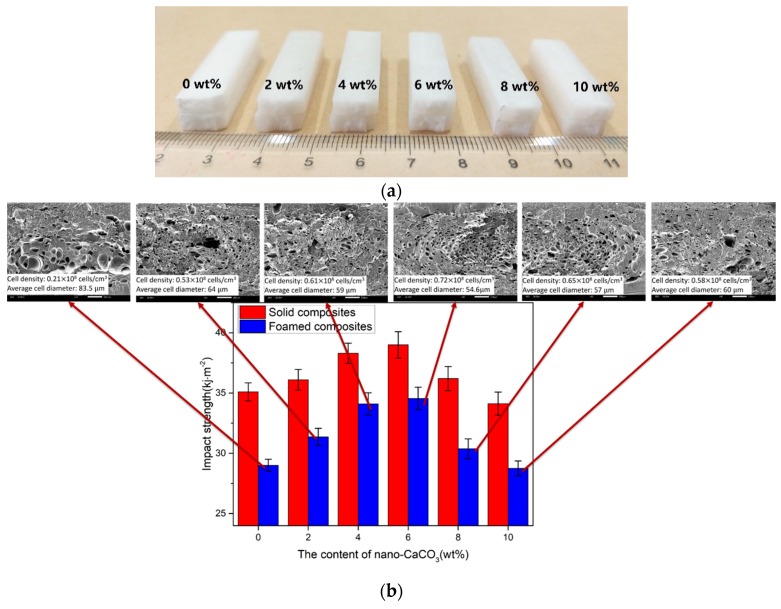
The results of the izod impact tests: (**a**) the samples after impact tests; (**b**) the impact strength.

As shown in Figure 8, the impact strength of the solid and foamed composites increased first and then decreased with the addition of nano-CaCO_3_. With the addition of nano-CaCO_3_, the change of impact strength of the foamed composites was more obvious. As shown in Figure 8b, there were more concentrated and uniform cells in the vertical section when the content of nano-CaCO_3_ was 6 wt %. It is evident that more cells absorb more impact energy. When the content of nano-CaCO_3_ was over 6 wt %, the efficiency of nucleation declined and there was less cell generating, so the impact strength decreased. When the content of nano-CaCO_3_ was 10 wt %, the impact strength of the samples was even smaller than those without the addition of nano-CaCO_3_.

As discussed above, it is evident that there are some effects of the microcellular structure on the mechanical properties. It is important to investigate the microcellular structure of the microcellular foam PP nanocomposites with different contents of nano-CaCO_3_.

### 3.3. Effect of the Content of Nano-CaCO_3_ on the Cells Structure

**Figure 9:**
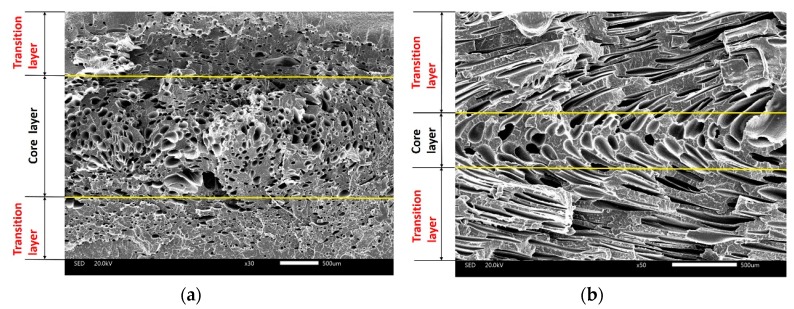
The distribution of cells of (**a**) vertical and (**b**) parallel sections.

As the nucleating agent, the addition of nano-CaCO_3_ affected the cell structure and distribution because there were more heterogeneous nucleations. The cells of vertical and parallel sections were investigated. The results showed that both the vertical and parallel sections were divided into three layers (two transition layers and one core layer), as shown in Figure 9.

From Figure 9a, it could be concluded that the cell size of the core layer was bigger than that of the transition layer, and that different cooling speeds caused this distribution. The high cooling speed of the transition layer left less time for the cells to grow up. The temperature of the core layer was higher, and there was enough time for the cells to grow up, so the size of the core layer was bigger than those of the transition layer.

From Figure 9b, the cell structures along the direction of the melt flow were totally different compared with the cells in the vertical section, because the cells that nucleated and grew in the period of filling deformed along the direction of melt flow. This phenomenon was also affected by the “fountain effect”. There was less area of the core layer, because of the settings of the process parameter. Through changing the process parameter, such as with the injection temperature and foaming time, the area of the transition layer could change.

#### 3.3.1. The Cell Structure of Vertical Section

(1) Core layer of Vertical Section

**Figure 10:**
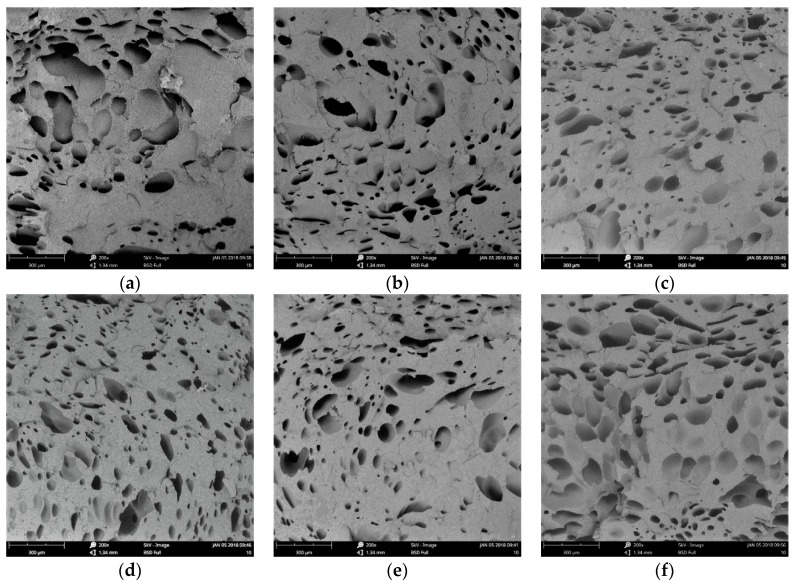
The results of the cell structure of the core layer of the vertical sections with different content of nano-CaCO_3_: (**a**) 0 wt %; (**b**) 2 wt %; (**c**) 4 wt %; (**d**) 6 wt %; (**e**) 8 wt %; (**f**) 10 wt %.

The cell structure of the core layer of the vertical sections with different contents of nano-CaCO_3_ are shown in Figure 10. It can be easily observed that there were many big cells in the microcellular foam neat PP (without nano-CaCO_3_), from Figure 10a. Because the neat PP with a low melt strength made it hard to control the cell structure, the cells coalesced easily when the cells grew. Without the addition of a nucleation agent, the major cell nucleation of neat PP was homogeneous nucleation, and it was not good for the distribution of the cells. With the addition of nano-CaCO_3_, the diameters of the cells decreased, and there were more cells generating. Research indicates that the cells nucleate in the boundary between the polymer matrix and the nano-CaCO_3_, and as the homogeneous nucleation agent, nano-CaCO_3_ can provide many nucleating sites [19]. There is both homogeneous and heterogeneous nucleation when the content of nano-CaCO_3_ is 2 wt % and 4 wt %, as shown in Figure 10b,c. When the content of nano-CaCO_3_ is 6 wt %, the average diameter of the samples was the smallest, the cell density of the samples was the biggest, and the distribution of the cells was also great, because the major way of nucleation was heterogeneous nucleation, and the cells nucleated and grew evenly, as shown in Figure 10d. This could improve the mechanical properties of the samples. With the continuous addition of nano-CaCO_3_, the cell structure got worse, as shown in Figure 10e,f. It is obvious that there were many large-sized cells, because the nano-CaCO_3_ conglomerated as the nano-CaCO_3_ was too much for a certain amount of compatilizer. It caused the uneven distribution of cells and it was easier for the cells to coalesce. The big and uneven distribution of the cells and nano-CaCO_3_ may cause the mechanical properties to get worse, as discussed above.

**Figure 11:**
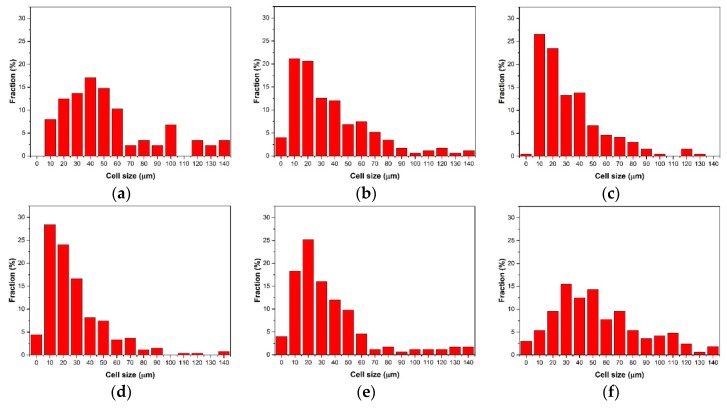
The cell size distribution of the core layer of the vertical sections with different contents of nano-CaCO_3_: (**a**) 0 wt %; (**b**) 2 wt %; (**c**) 4 wt %; (**d**) 6 wt %; (**e**) 8 wt %; (**f**) 10 wt %.

The cell size distribution described the cells’ proportion in different ranges of cell size. As shown in Figure 11a, the cell size distribution of the samples with 0 wt % nano-CaCO_3_ was bad. There were many large-sized cells with diameters larger than 100 μm; the amount of cells where the diameters were from 10 μm to 60 μm is large. The range of the cell size distribution was large. With the addition of nano-CaCO_3_, the cell distribution improved. The number of large cells decreased, and the cells had a concentrated distribution, as shown in Figure 11b–d. The cell size distribution of the samples with 6 wt % nano-CaCO_3_ was the optimum, as shown in Figure 11d. When the content of nano-CaCO_3_ was more than 6 wt %, the cell distribution got worse, as shown in Figure 11e,f. There were more large-sized cells with diameters larger than 100 μm.

**Figure 12:**
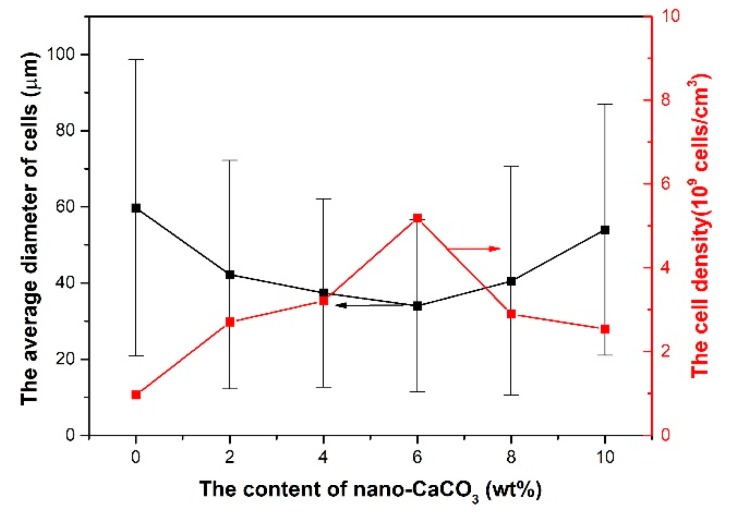
The average cell diameter and cell density of the core layer of the vertical sections with different contents of nano-CaCO_3_.

The average cell diameter and cell density can be calculated with Image Pro Plus. The results of the average cell diameter and cell density are shown in Figure 12.

The average diameter of the cells decreased with the addition of nano-CaCO_3_ and increased when the content of nano-CaCO_3_ was more than 6 wt %. The cell density increased with the addition of nano-CaCO_3_ and decreased when the content of nano-CaCO_3_ was more than 6 wt %. The best cell structure was found, with an average diameter of 34 μm and cell density of 5.18 × 10^9^ cells/cm^3^, when the content of nano-CaCO_3_ was 6 wt %. The reason for the cell structure changing in this way is that as the nucleating agent, the nano-CaCO_3_ can affect the cells’ nucleating and their distribution.

**Figure 13:**
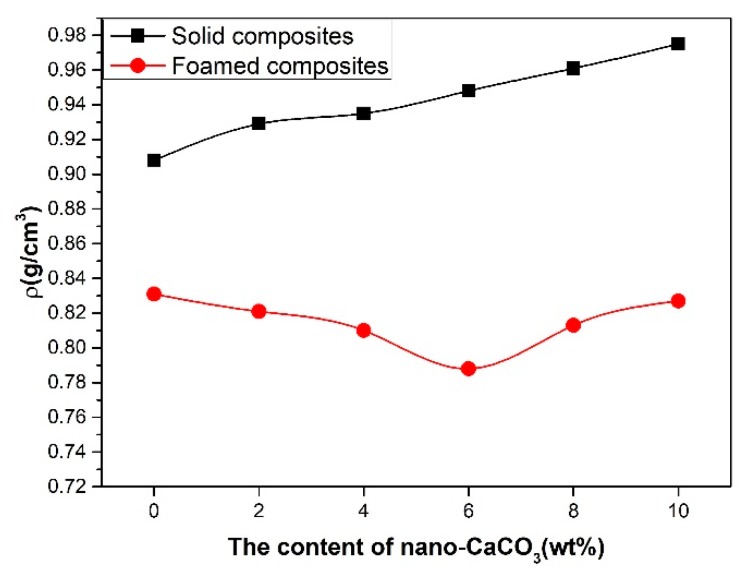
The density of solid composites and foamed composites.

As shown in the Figure 13, the density of the foamed composites decreased and then increased with the content of nano-CaCO_3_. It was opposite to the change of the cell density, because more cells mean that less gas is separated out. The content of the foamed agent was same in this work, so more cells helped in reducing the density of the foamed composites. When the amount of nano-CaCO_3_ was over 6 wt %, there were less polymers for foaming, and the addition of nano-CaCO_3_ did improve the mass of composites, so the density increased.

(2) Transition Layer of Vertical Section

**Figure 14:**
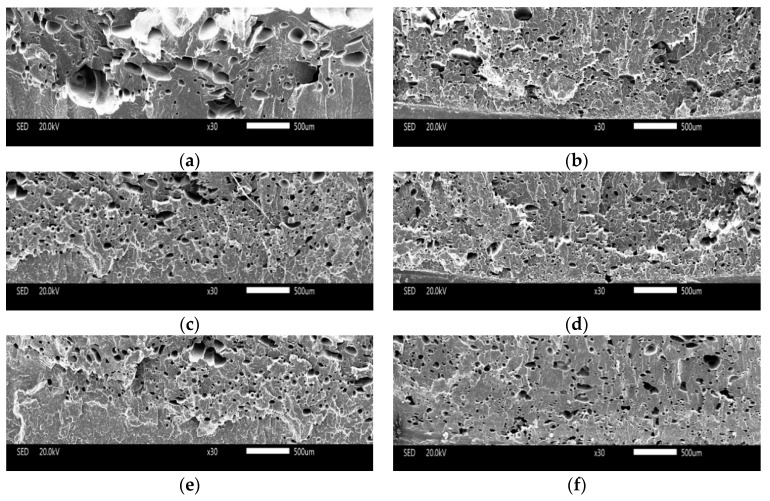
The cells structure of the transition layer of the vertical sections with different contents of nano-CaCO_3_: (**a**) 0 wt %; (**b**) 2 wt %; (**c**) 4 wt %; (**d**) 6 wt %; (**e**) 8 wt %; (**f**) 10 wt %.

The cell structure of the bottom transition layer of PP with different contents of nano-CaCO_3_ is shown in Figure 14 (the bottom transition layer is similar to the top transition layer; one transition layer has been investigated in this paper).

There were some big cells from the transition layer of the vertical section of the neat PP, as shown in Figure 14. It can be concluded that there is no obvious difference in the cell size when the content of nano-CaCO_3_ changes from 2 wt % to 10 wt %. The cell sizes of the transition layer were smaller to those of the core layer, because the cooling speed of the transition layer was high and took less time to grow the cells. As shown in Figure 14, the cell sizes were smaller when the cells were closer to the mold (the bottom of the figure is the mold).

**Figure 15:**
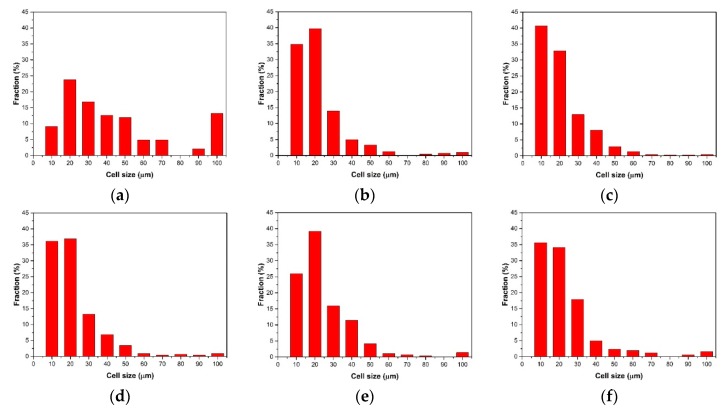
The cell size distribution of the transition layer of the vertical sections with the different contents of nano-CaCO_3_: (**a**) 0 wt %; (**b**) 2 wt %; (**c**) 4 wt %; (**d**) 6 wt %; (**e**) 8 wt %; (**f**) 10 wt %.

From Figure 15, it is obvious that the cell size distribution of the transition layer of microcellular foam PP without the addition of nano-CaCO_3_ is different than the others.

The cell size was more scattered in the transition layer of the neat PP, as shown in Figure 15a, while the cell size was more concentrated in the transition layer of PP with the addition of nano-CaCO_3_, as shown in Figure 15b–f.

**Figure 16:**
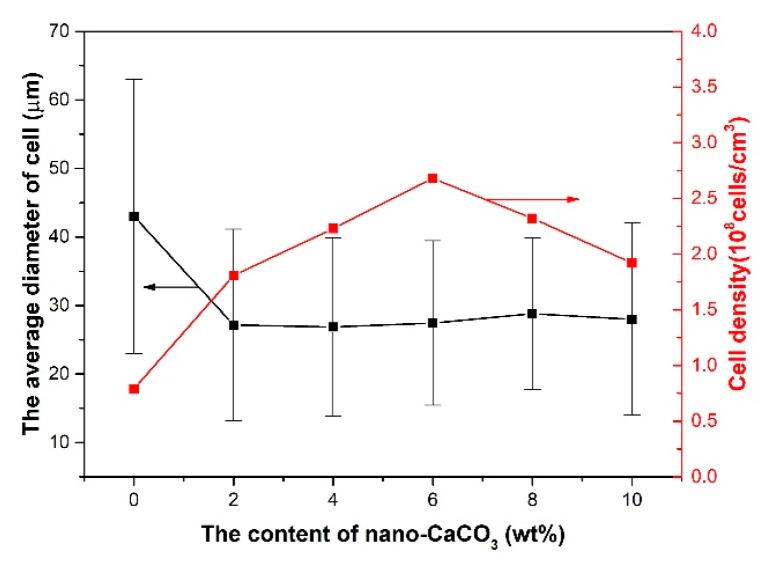
The average cell diameter and cell density of the transition layer of the vertical sections with different contents of nano-CaCO_3_.

As shown in Figure 16, the average cell diameter of the neat PP is large, because without the addition of nano-CaCO_3_, the crystallization of PP was homogeneous. The time taken for the homogeneous crystallization was longer than for the heterogeneous crystallization, and there was more time for cells to grow. The way in which the cells were nucleated was homogeneous nucleation, the cells were unevenly distributed. In addition, the low melt strength led to the cells’ coalescence. With the addition of nano-CaCO_3_, the average cell diameter decreased, and there was no evident difference in the average cell diameter with the further addition of nano-CaCO_3_. The temperature of the transition layer was lower than the core layer, and cells did not have enough time to grow up. The melt strength increased because of the addition of nano-CaCO_3_. There were less cells that experienced coalescence, so the cell diameters were the similar. The way in which the cell density changed is similar to that of the core layer.

#### 3.3.2. The Cell Structure of Parallel Section

(1) Core Layer of Parallel Section

**Figure 17:**
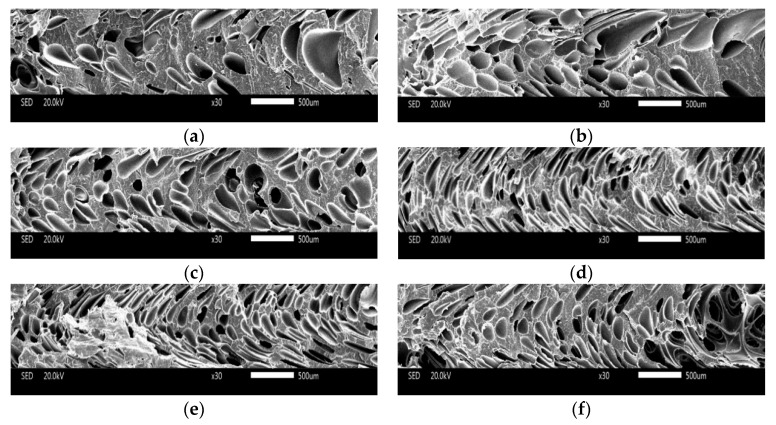
The cells structure of core layer of parallel sections with different content of nano-CaCO_3_: (**a**) 0 wt%; (**b**) 2 wt%; (**c**) 4 wt%; (**d**) 6 wt%; (**e**) 8 wt%; (**f**) 10 wt%.

It can be found that the cells were irregular and unevenly distributed without addition of nano-CaCO_3_, as shown in Figure 17. Without a nucleating agent, the low melt strength caused the cells to coalesce and the homogeneous nucleation of the cells caused the uneven distribution of cells. With the addition of nano-CaCO_3_, the cells were more regular and the cells were more dense. When the content of nano-CaCO_3_ was 6 wt%, the cell density was biggest and the cell sizes were the smallest. Firstly, the addition of nano-CaCO_3_, as the nucleating agent of the cells, improved the cell structure. Also, the addition of nano-CaCO_3_ improved the melt strength, which led to less cells coalescence. So, the cells were more regular and intensive.

**Figure 18:**
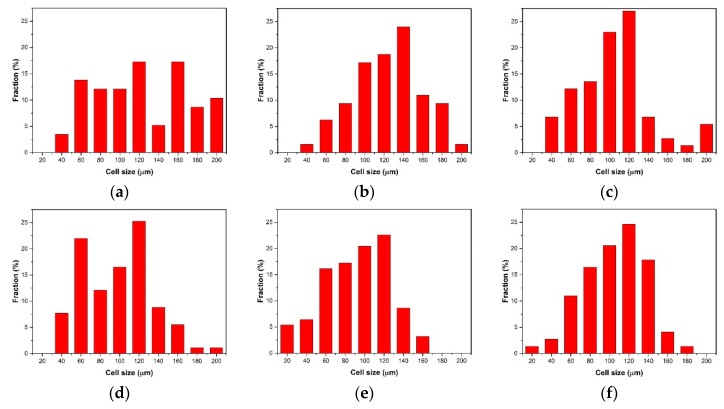
The cell size distribution of the core layer of parallel sections with different contents of nano-CaCO_3_: (**a**) 0 wt %; (**b**) 2 wt %; (**c**) 4 wt %; (**d**) 6 wt %; (**e**) 8 wt %; (**f**) 10 wt %.

As shown in Figure 18a, the cell sizes were not well-distributed, and there were many big cells in the core layer of the parallel section. With the addition of nano-CaCO_3_, there were more cells with sizes from 100 to 120 μm, as shown in Figure 18b–f. In addition, the cell sizes were the similar in Figure 18b–f. This could also could be seen from the SEM of the core layer of the parallel section.

**Figure 19:**
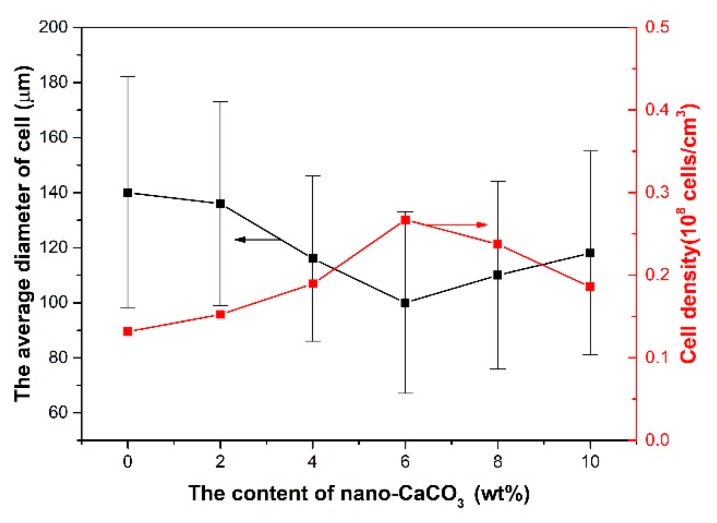
The average cell diameter and cell density of the core layer of parallel sections with different contents of nano-CaCO_3._

As for the way in which the average cell diameter and cell density change, the addition of nano-CaCO_3_ was similar to that in the vertical section, as shown in Figure 19. The average diameter of cells decreased with the addition of nano-CaCO_3_, and increased when the content of nano-CaCO_3_ was more than 6 wt %; and the trend of cell density change was opposite. When the content of nano-CaCO_3_ was 6 wt %, the cell density was at its biggest (0.267 × 10^8^ cells/cm^3^), and the average cell diameter was the smallest (100 μm). The main reason is that nano-CaCO_3_ improved the nucleation of cells and improved the melt strength. There were less cells coalescence and more well-distributed cells.

(2) Transition Layer of Parallel Section

**Figure 20:**
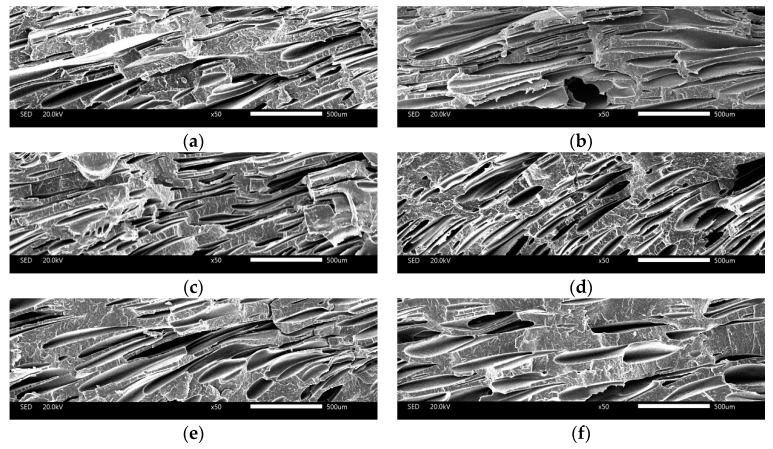
The cell structure of the transition layer of parallel sections with different contents of nano-CaCO_3_: (**a**) 0 wt %; (**b**) 2 wt %; (**c**) 4 wt %; (**d**) 6 wt %; (**e**) 8 wt %; (**f**) 10 wt %.

As shown in Figure 20, the cell structure of the transition layer of the parallel section is totally different to that of the vertical section.

**Figure 21:**
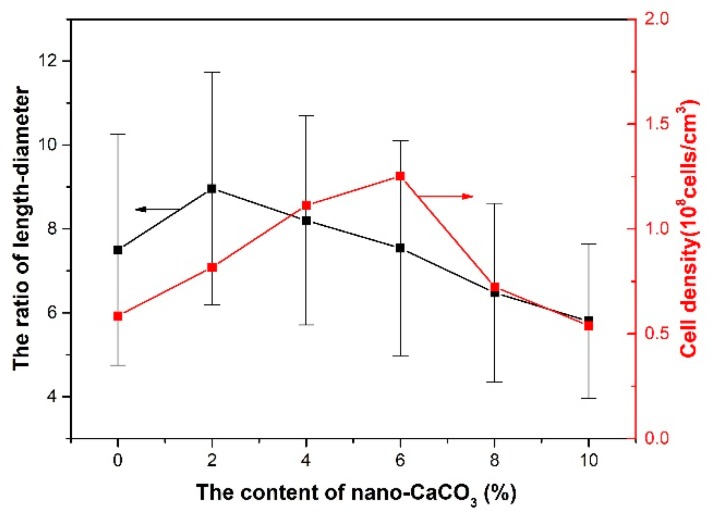
The average ratio of length–diameter and cell density of the transition layer of parallel sections with different content nano-CaCO_3_.

The cells that nucleated and grew up in the period of filling deformed under the shear stress. The cells changed to an ellipse and the deformation of the cells was along the melt flow. The average ratio of length–diameter of the transition layer cell can be used to characterize the deformation to some degree. The large ratio of length–diameter means that the deformation is large. This paper did not investigate the average cell diameter of the transition layer of the parallel sections. It is meaningless to investigate the average cell diameter because of deformation. The results of the average ratio of length–diameter and cell density are shown in Figure 21.

When the content of nano-CaCO_3_ was 2 wt %, the average ratio of length–diameter increased compared with the one without the addition of nano-CaCO_3_, because without addition of nano-CaCO_3_, the cell density was smaller, the cell walls were bigger, and the ability of resistance to deformation was bigger. With the continuous addition of nano-CaCO_3_, the melt strength increased and the ability of resistance to deformation improved. The deformation of the transition layer cell decreased and the average ratio of length–diameter decreased, too.

## 4. Conclusions

The effects of the nano-CaCO_3_ content on the crystallization behavior, mechanical properties, and cell structure of microcellular foam PP nanocomposites were investigated. The thermal stability and crystallinity increases with the increase of the nano-CaCO_3_. The mechanical properties increased first and then decreased with addition of nano-CaCO_3_, because of the synergistic effect of the cell structure and nano-CaCO_3_. The cell structures of the parallel and vertical sections were used to characterize the cell structure of the whole sample. Both the cells of parallel and vertical sections were divided into three parts (two transition layers and one core layer). The addition of nano-CaCO_3_ could improve the cell structure. When the content of nano-CaCO_3_ was 6 wt %, the cell structures both in the parallel and vertical sections were the best.

In conclusion, the addition of nano-CaCO_3_ can improve the crystallization behavior, mechanical properties, and cell structure of microcellular foam PP. When the content of nano-CaCO_3_ was 6 wt %, the mechanical properties and cell structure were optimum.

## Figures and Tables

**Table 1:** Comparison of the fusion and crystallization properties of nanocomposites.

Content of nano-CaCO_3_ (wt%)	T_m_ (°C)	T_c_ (°C)	H_m_ (J/g)	Crystallinity (%)
0	169.62	126.29	80.83	38.67
2	169.35	128.85	78.92	37.76
4	169.00	129.57	85.26	40.79
6	168.61	129.46	86.56	41.42
8	169.98	130.63	87.61	41.92
10	169.39	131.13	87.83	42.23

**Table 2:** Comparison of Thermogravimetric analysis (TGA) properties of nanocomposites.

Content of Nano-CaCO_3_ (wt%)	T_d_ (°C)	DTG at T_d_ (%/min)	Residue at 550 °C (%)	Residue at 800 °C (%)
0	454.6	−30	0.3	0.1
2	456.3	−28.4	2.5	1.4
4	452.9	−20.6	5.1	2.8
6	454.4	−23.5	6.4	3.9
8	454.3	−22.8	8.7	5.2
10	452.4	−21.9	10.8	7.2
